# Design of a Spark Big Data Framework for PM_2.5_ Air Pollution Forecasting

**DOI:** 10.3390/ijerph18137087

**Published:** 2021-07-02

**Authors:** Dong-Her Shih, Thi Hien To, Ly Sy Phu Nguyen, Ting-Wei Wu, Wen-Ting You

**Affiliations:** 1 Department of Information Management, National Yunlin University of Science & Technology, Douliu 64002, Taiwan; portraits1129@gmail.com (T.-W.W.); s87panner@gmail.com (W.-T.Y.); 2 Faculty of Environment, University of Science, 227 Nguyen Van Cu Street, District 5, Ho Chi Minh City 700000, Vietnam; tohien@hcmus.edu.vn (T.H.T.); nlsphu@hcmus.edu.vn (L.S.P.N.); 3 Vietnam National University, Linh Trung Ward, Thu Duc District, Ho Chi Minh City 700000, Vietnam

**Keywords:** air pollution, PM_2.5_ predictions, machine learning, Spark, ensemble model, big data

## Abstract

In recent years, with rapid economic development, air pollution has become extremely serious, causing many negative effects on health, environment and medical costs. PM_2.5_ is one of the main components of air pollution. Therefore, it is necessary to know the PM_2.5_ air quality in advance for health. Many studies on air quality are based on the government’s official air quality monitoring stations, which cannot be widely deployed due to high cost constraints. Furthermore, the update frequency of government monitoring stations is once an hour, and it is hard to capture short-term PM_2.5_ concentration peaks with little warning. Nevertheless, dealing with short-term data with many stations, the volume of data is huge and is calculated, analyzed and predicted in a complex way. This alleviates the high computational requirements of the original predictor, thus making Spark suitable for the considered problem. This study proposes a PM_2.5_ instant prediction architecture based on the Spark big data framework to handle the huge data from the LASS community. The Spark big data framework proposed in this study is divided into three modules. It collects real time PM_2.5_ data and performs ensemble learning through three machine learning algorithms (Linear Regression, Random Forest, Gradient Boosting Decision Tree) to predict the PM_2.5_ concentration value in the next 30 to 180 min with accompanying visualization graph. The experimental results show that our proposed Spark big data ensemble prediction model in next 30-min prediction has the best performance (R^2^ up to 0.96), and the ensemble model has better performance than any single machine learning model. Taiwan has been suffering from a situation of relatively poor air pollution quality for a long time. Air pollutant monitoring data from LASS community can provide a wide broader monitoring, however the data is large and difficult to integrate or analyze. The proposed Spark big data framework system can provide short-term PM_2.5_ forecasts and help the decision-maker to take proper action immediately.

## 1. Introduction

In recent years, with rapid economic development, air pollution has become increasingly serious, causing many negative effects on health, environment and medical costs. The World Health Organization’s (WHO) report also mentions for about three-quarters of the world’s population, the air pollution concentration values of living environments exceeds those specified by the WHO, and indoor and outdoor air pollution causes about 7 million premature deaths every year [1]. Air pollution can cause many diseases and negatively affect human health. Martinelli, Olivieri and Girelli [2] have pointed out that exposure to fine suspended particulates (PM_2.5_) can easily lead to an increase in the incidence of cardiovascular diseases. IARC [3] also mentioned that exposure to outdoor air pollution can cause lung cancer and increase the risk of bladder cancer and breast cancer. One study by Hwang et al. [4] shows that air pollution will increase hospitalization rates, leading to an increase in medical costs.

Therefore, some scholars have conducted research to find out the factors related to air pollution [5]. In terms of air quality prediction, there are also many studies based on different methods, such as the use of neural networks to predict three days in advance the concentration of SO_2_, CO, and PM_10_ in Besiktas District, Istanbul [6]. Li, Huang, and Luo [7] use computer vision technology to analyze photos obtained from social media to establish a correlation between the degree of smog on the photo and official PM_2.5_ data, to predict PM_2.5_ concentration value. Lee et al. [8] use the random forest method and the weather data and road information generated by the city sensing system to predict the AQI value.

Nevertheless, many studies on air quality in Taiwan are based on the government’s official air quality monitoring stations, which cannot be widely deployed due to high cost constraints [9]. Therefore, it is very important for the public to provide small-scale, low-cost and real-time air quality information that can penetrate into people’s living areas [10]. However, the update frequency of government monitoring stations is once an hour, and it is impossible to capture short-term PM_2.5_ concentration peaks. The data source used in this study is provided by the LASS open source community, which has more than 1000 sensing stations and the data update frequency is every five minutes. This study aims to provide an immediately predicted PM_2.5_ concentration value in the next 30 min to 180 min. In the face of big data and real-time challenges, cloud platforms have shown fault tolerance, scalability and load balancing characteristics [11]. Therefore, this study tries to build all the processes of the real-time prediction model on the cloud big data platform, and use the advantages such as speed, flexibility and stability to solve the difficulties and challenges encountered from servers.

Forecasting the PM_2.5_ level helps the public and the hospital to take precautionary actions immediately. This study aims to use the PM_2.5_ sensor data provided by the open source community Location Aware Sensing System (LASS), and use the Spark big data computing framework and machine learning algorithms to build a real-time prediction model, perform real-time prediction of PM_2.5_ concentration value in order to achieve the purpose of PM_2.5_ early warning and air pollution monitoring. Short-term PM_2.5_ predictions become more accurate with the increase of sensors, but short-term predictions are still affected by many unexpected situations and limit the accuracy of the predictions [12]. The LASS community has a huge data set, and the system can still reach an efficient level through the calculation of big data. The quality of air pollution in Taiwan has not been as good as expected for many years. Short-term forecasts can ensure that information is easily distributed, and relevant decision units can use the information to formulate response plans.

To the best of our knowledge, this is the first Big data Spark predicting solution to deal with large-scale, high-speed and streaming problems. The main contributions of this paper are as follows. (1) This study combines the pollutant components in different time periods into the input variables for prediction. It is one of the few research methods used in most PM_2.5_ concentration prediction studies. (2) Through different window sizes, test which time interval is most beneficial to the prediction result. (3) The sensors used by the LASS community are also the most widely distributed sensor project in the study. (4) To quickly collect and process a large amount of data. Experimental results show that our proposal outperforms the single machine learning model in terms of accuracy. Our method also reduces the time spent in the prediction stage and the memory consumption by using Spark platform.

The organization of the paper is as follows: Section 2 presents a Literature Review. Section 3 describes the Framework of Proposed Spark Big Data Model and Section 4 describes the data set information that is used for experimentation and evaluation. In Section 5, discussions of the evaluation are detailed with the results obtained. Finally, the paper concludes with the future scope in Section 6.

## 2. Literature Review

### 2.1. Air Pollution

Air pollution means that there are one or more pollutants in the air that are harmful to human health and property. Common air pollutants include carbon monoxide (CO), sulfur dioxide (SO_2_), nitrogen oxides (NOx), ozone (O_3_), particulate matter (PM), etc. Among them, the main component that is seriously harmful to human health is fine suspended particulates (PM_2.5_) [13].

PM_2.5_ is one of the main components of air pollution, which can carry harmful substances such as pathogenic microorganisms and chemical pollutants, deep into the alveoli, bronchioles, and blood circulatory system, causing diseases such as pneumonia, cardiovascular disease and cancer, and has a significant impact on human health, thus it is considered to be more harmful than PM_10_ [14]. As early as the WHO’s report on global health risks published in 2009, urban air pollution was mentioned as the 14th leading cause of death in 2004 [15]. In view of the direct impact of air pollution on health, many studies have confirmed that air pollution is related to a variety of diseases. Currently known related diseases include respiratory diseases, cardiovascular diseases, diabetes and cancer, etc. [16,17,18,19,20]. Brook et al. [21] pointed out that short-term exposure to a high PM environment will cause an increase in acute cardiovascular mortality, while long-term exposure to a high PM environment will reduce overall life expectancy. Krämer et al. [22] have confirmed that traffic-related air pollution is a risk factor affecting type 2 diabetes. In recent years, studies in Taiwan have also confirmed that long-term exposure to PM_2.5_ can increase the risk of liver cancer [23]. In addition, related work of Air Pollution is described below:

- Correlation of Air Pollutants

Zhang, Ni and Ni [24] used multifractal descended cross-correlation analysis (MF-DCCA) to analyze the relationship with PM_2.5_ and temperature, air pressure, relative humidity and wind speed, and found cross-correlation. Lu, Xu, Yang and Zhao [25] used grey system correlation analysis and linear regression to explore the temporal and spatial changes of PM_2.5_ in China from 1998 to 2014, and found that the concentration of PM_2.5_ in China is significantly different depending on the spatial distribution. Furthermore, China’s socio-economic and meteorological conditions have a significant impact on PM_2.5_. There are also studies on the correlation analysis and short-term prediction of Beijing PM_2.5_ based on data from various sources such as social media, average relative humidity, regional average rainfall, CO, NO_2_ and PM_10_. In a correlation study, the analysis was based on the Back Propagation Neural Network, and the results found that the average wind speed value, social media data, CO, NO_2_ and PM_10_ five factors, and the PM_2.5_ concentration value show a high degree of mathematical correlation [26].

- Air quality prediction

There are also many studies based on different machine learning methods in air quality prediction. For example, air quality prediction using neural networks is used in the two regions of Thessaloniki, Greece and Helsinki, Finland. They use principal component analysis (PCA) and neural networks to select the input features of the prediction model to achieve the purpose of predicting PM_2.5_ and PM_10_ [27]. Yao, Lu and Jiang [28] established a PM_2.5_ estimation model using neural network through ground monitoring data from March to May 2008 in northern China. The results show that the estimation efficiency of PM_2.5_ daily average value based on the neural network is higher than that of the multiple regression method. Other scholars also used the Hidden Markov Model to predict the average daily PM_2.5_ concentration value in Northern California one day in advance [29]. Wang, Liu, Qin and Zhang [30] used improved support vector machines and neural networks to predict PM_10_ and SO_2_ in Taiyuan city, China. Some scholars also use support vector machines as prediction models, and use partial least squares to select data to predict CO concentration [31]. Yu, Yang, Yang, Han and Move [32] use the random forest method to predict the AQI value and have good prediction accuracy. In order to improve the air quality measurement problems in many developing countries that lack monitoring stations, some studies have taken Beijing, Shanghai and Wuhan as study areas. Through the proposed combination of good and bad social media metrics and seven meteorological factors, and the use of Gradient Tree Boosting (GTB) machine learning methods, the value of the city’s AQI can be inferred [33].

- Sensor of Air quality

Since professional air quality sensor stations are very large and expensive, they cannot be built extensively and densely, which has caused many scholars to conduct studies on low-cost miniature sensors, there is a large-scale PM_2.5_ sensor deployment project in Taiwan, in cooperation with the government, the public, academic institutions etc. As of May 2015, more than 2500 devices have been installed in Taiwan and other countries, and the source code is open, so that people who want to contribute to the system can easily participate, open source the acquired sensor data, and update it in real time in every five minutes, so as to achieve a participatory urban PM_2.5_ monitoring system [34]. Gao et al. [35] developed Mosaic, a low-cost mobile sensing system for urban air quality, and set up air quality monitoring nodes on buses, increasing the range of the system to obtain a larger range for each node, so that a small number of sensor stations can cover a wide range and reduce costs. Related research on air pollution is summarized as shown in Table 1.

### 2.2. Input and Output Variables of PM_2.5_ Prediction Model

The variables associated with air pollution can be found in previous studies, there are weather, traffic, air pollutants, social media, etc. Variables are described below.

- Traffic

Studies have pointed out that the number of self-use cars and scooters has increased significantly, causing traffic air pollution to become the main component of overall air pollution [38]. The CO, NOx and SO_2_ in the gas emitted by vehicles are all important sources of PM_2.5_ [39]. Therefore, the increase in the number of self-use vehicles is considered to be one of the reasons for the increase in PM_2.5_ concentrations [40,41]. However, the sensors in this study did not provide traffic data. Dhyani et al. [42] used road type and road alignment to predict PM_2.5_ from urban highway corridors, and Kwiecień and Szopińska [43] combined average daily traffic (AADT), carbon monoxide emissions for every road section and other variables to predict the degree of air pollution. Walsh [44] investigate global progress in controlling the motor vehicle contribution.

- Weather

Many studies mentioned that rainfall and wind direction are negatively correlated with air pollution values [7,19,45]. Sun et al. [29] indicated that the temperature is related to PM_2.5_ forecast. This study uses Temperature and Humidity as the input variables of the PM_2.5_ prediction model in terms of weather variables. Pak et al. [46] used the highest and lowest temperature of the daily into the input variables. Xing et al. [47] incorporated mean atmospheric pressure into input variables to make predictions. Polichetti et al. [48] also pointed out that the observed values of relative humidity and wind speed are good indicators for predicting the concentration of PM_2.5_.

- PMs

Particulate Matter (PM) is one of the main indicators of air pollution, and PM_2.5_ is the main component of air pollution. The main purpose of this study is to predict PM_2.5_, so we selected PM_10_ with an aerodynamic diameter less than or equal to 10 microns, PM_2.5_ less than or equal to 2.5 microns and PM_1_ less than or equal to 1 micron are used as input variables in the model. Furthermore, PM_2.5_ is used as the input and output variables in the prediction model.

### 2.3. Machine Learning

Machine learning belongs to a branch of artificial intelligence. It is a data analysis method that enables machines to automatically learn from data through algorithms. Training through a large amount of data, the machine analyzes and finds rules from it, and improve its performance through experience, so as to obtain the ability to automatically judge or predict.

In the study of PM_2.5_ concentration value prediction using machine learning methods, it is pointed out that random forests are explanatory to the results, can show the importance of each prediction variable, and can strike a balance between prediction accuracy and computational cost [49]. In addition, the PM_2.5_ concentration value prediction is suitable for the use of regression methods, and the ensemble model can improve the accuracy. Based on the above, this study selects Linear regression, Gradient-boosted tree regression and Random forest regression in Spark’s MLlib. Combine these three algorithms to perform ensemble learning method to produce predictive model.

#### 2.3.1. Ensemble Learning

**Figure 1:**
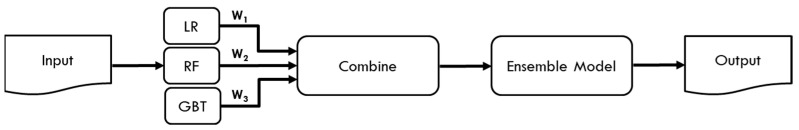
Flowchart of ensemble learning.

The basic idea of ensemble learning is to combine multiple machine learning algorithms. It is expected that the result of the ensemble will be better than a single machine learning method. The goal is to train and combine different models to obtain better performance than any single model [50]. The method is to train multiple models using machine learning methods, and adjust all the models through weight adjustment, voting, averaging etc., to produce more accurate results. This study will combine three machine learning algorithms through ensemble learning to form an ensemble model as a PM_2.5_ concentration value prediction model. The process of ensemble learning in this study is shown in Figure 1:

#### 2.3.2. Spark MLlib

Apache Spark is an open source big data computing framework developed using Scala. It was originally developed by AMPLab of the University of California, Berkeley, was open sourced in 2010, and has now become a top project in the Apache Software Foundation. In addition to the advantages of fast computing, Spark can also be flexibly written in four programming languages such as Scale, Java, Python and R. It is also compatible with the Hadoop platform. A variety of functions can be combined to provide a more convenient way for development developers. The Spark has a machine learning library MLlib, which is suitable for iterative machine learning operations, and has the characteristics of being suitable for real-time situations. Therefore, it fits the context of this study using machine learning algorithms for real-time prediction.

Machine Learning Library (MLlib) is Spark’s open source distributed machine learning framework, which was developed in 2012 as part of the MLBase project [51]. It was open sourced in September 2013 [52]. Usually machine learning algorithms need to perform a large number of iterative operations, and the design of Spark can improve the performance of executing multiple iterations. Because of its memory-based execution of operations, it is naturally capable of iterative operations, which is also in line with the characteristics of many machine learning algorithms [52]. Moreover, MLlib can be easily combined with Spark Streaming, Spark SQL and GraphX naturally.

MLlib is a part of MLBase, including classification, clustering, regression, collaborative filtering, feature extraction, dimension reduction and other machine learning algorithms and tools. It mainly provides the following functions:

(1) ML Algorithms: Provide machine learning algorithms such as classification, clustering, regression, and collaborative filtering.
(2) Featurization: includes methods such as feature extraction, feature selection and dimension reduction.
(3) Pipelines: Provide tools for constructing, evaluating and adapting ML Pipelines.
(4) Persistence: Provides the ability to persist machine learning models, which means that machine learning models can be saved and loaded, making model development more convenient.
(5) Utilities: Including data processing, statistics and other tools.

After Spark2.0 version, Spark’s main machine learning API has turned to the DataFrame-based API in the spark.ml package as the main provider. Compared with the RDD-based API, it can be combined with more functions, such as the operation of SQL, which is more convenient. It is also more suitable for building ML Pipelines including a series of machine learning processes such as data processing, model training and testing.

### 2.4. Google Cloud Platform

Cloud computing enables IT to be provided as a public service to meet the increasing demand for information services, so it has become an important technology. Many cloud providers have appeared to provide more convenient cloud services. At present, the main cloud service providers are Google’s Google Cloud Platform (GCP), Microsoft’s Microsoft Azure, Amazon’s Amazon Web Services (AWS), and so on. Google has its own data center in Changhua Coastal Industrial Park, Taiwan, so GCP has advantages in speed, convenience and data security for users in Taiwan. And GCP uses minutes instead of hours to provide a more flexible pricing standard. In terms of machine learning, GCP can use the TensorFlow architecture that has been used in many Google products such as Google Photo Album and Google Cloud Speech to build models. TensorFlow is an open source machine learning software library. It has libraries for many users in the field of machine learning and artificial intelligence. Due to the large number of users, many resources can be searched, and it is also under continuous development.

In addition, the visualization tool used in this study is PlotDB developed by Taiwan, which provides data visualization services. It not only has basic line charts, pie charts, bar charts, but also various special chart styles such as maps of countries and interactive charts for users to choose. Users can also create a chart online, or view the JavaScript, HTML, and CSS code of the chart. PlotDB also provides APIs for users to use them flexibly according to their needs.

## 3. Methodology

### 3.1. Spark Big Data Framework

**Figure 2:**
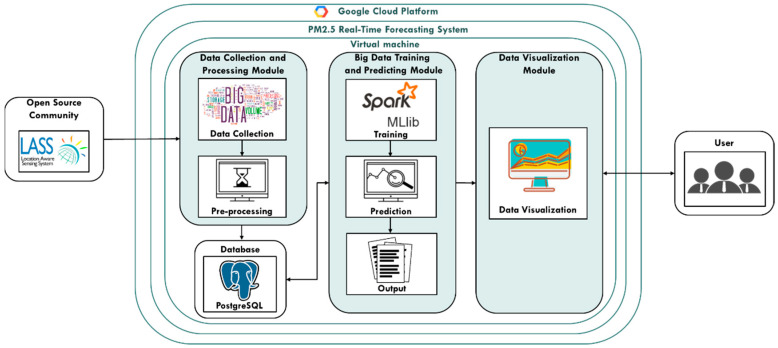
Spark big data Framework.

The Spark big data Framework proposed by this study is mainly divided into Data Collection and Processing Module, Big Data Training and Predicting Module and Data Visualization Module. First, the data collection and processing module will collect PM_2.5_ prediction-related data from the open source community LASS, and perform data pre-processing, storing it in the PostgreSQL database. Then, the big data training and predicting module is used to train and build the PM_2.5_ concentration value prediction model, and use the established prediction model to predict PM_2.5_ in real time. The prediction results are passed into the data visualization module and drawn into a PM_2.5_ concentration value distribution map as shown in Figure 2:

#### 3.1.1. Data Collection and Processing Module

The data collection and processing modules of this study are subdivided into “Data Collection Module” and “Pre-processing Module”. In the data collection module, the Timer mechanism periodically calls the data collection function according to the frequency required for data collection. Timer calls the PM_2.5_ data collection functions every five minutes, so the real-time collection of research data can be carried out through the Timer mechanism.

**Figure 3:**
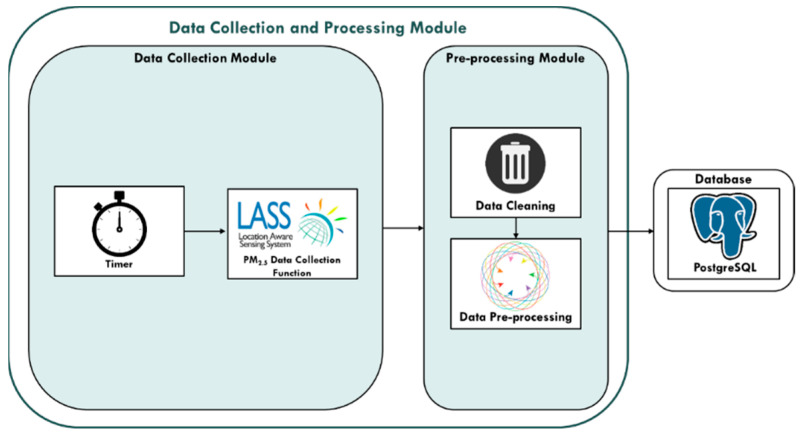
Data Collection and Processing Module.

In the pre-processing module, sequentially data cleaning from the original data, and data is processed for Missing Value, Outlier, and Noise. Then, the processed data is saved into the PostgreSQL database for other processes. The data collection and processing module diagram of this research is shown in Figure 3:

#### 3.1.2. Big Data Training and Predicting Module

**Figure 4:**
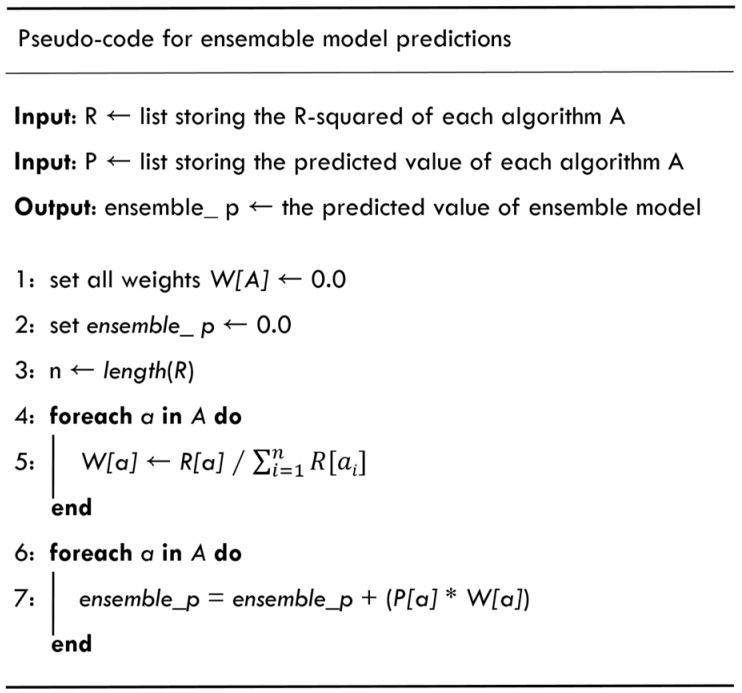
Pseudocode of ensemble learning.

In the Spark big data training module, the pre-processing the collected historical data of PM_2.5_ air quality to generate the output and input variables of the model is conducted first, and then the model is built for training and testing stage. In the model training stage, using the machine learning library MLlib provided by Spark, three different machine learning methods, Linear Regression (LR), Random Forest (RF), and Gradient Boosting Decision Tree (GBT) are chosen. Further, ensemble learning for model training and testing is used, combined with three different weights of the three algorithms to form an ensemble model. The weights of the three machine learning methods are adjusted through the value of the evaluation index R^2^. The detailed weighting method of the ensemble model and the pseudo code of the prediction method are shown in Figure 4 below.

**Figure 5:**
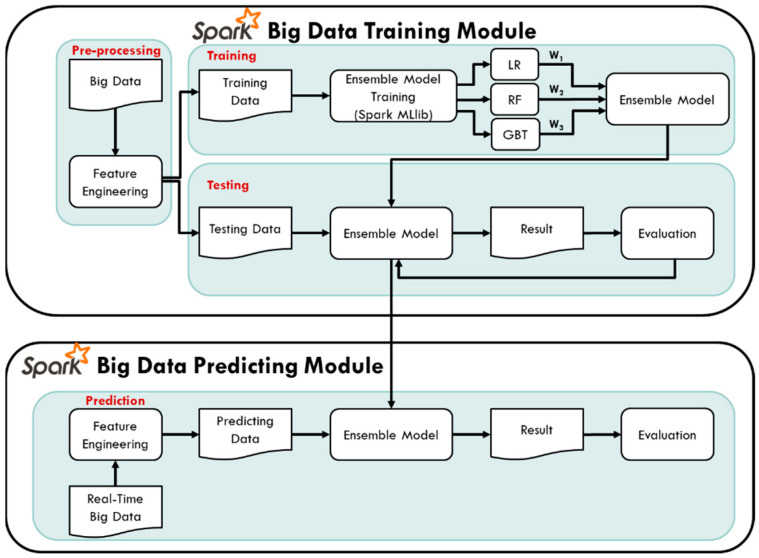
Training and testing of PM_2.5_ prediction model.

Finally, in the Spark big data prediction module architecture, feature engineering steps generate the data, and then are input into the ensemble model for real-time PM_2.5_ concentration prediction. The air quality machine training and prediction flowchart of this study is shown in Figure 5:

#### 3.1.3. Data Visualization Module

**Figure 6:**
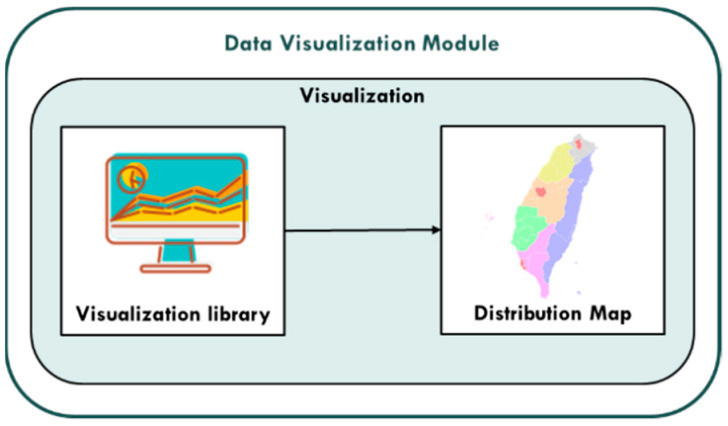
Data visualization module.

The PM_2.5_ concentration value prediction results of each monitoring station generated by the big data training and prediction module are used to visualize the PM_2.5_ concentration value of each region using the data visualization service provided by PlotDB. The data visualization module diagram of this research is shown in Figure 6:

## 4. Experimental Design

### 4.1. Data Source

#### 4.1.1. Open Source Community

The source of the data collected in this study is the Open Source Community, named LASS (Location Aware Sensing System), an open source and non-profit community that has deployed more than 2000 devices in Taiwan and is currently the largest monitoring PM_2.5_ deployment project [11]. Thousands of sensor stations can provide higher-density air quality data, which can complement the official Air Quality Index (AQI) values provided by the government. The PM_2.5_ related information of this study is obtained from the LASS open source community. The sensor type of LASS is EDIMAX AIRBOX, and the frequency is once every 5 min, the sensor data has 10 variables including date, time, device id, PM_2.5_ concentration value, PM_10_ concentration value, PM_1_ concentration value, temperature, relative humidity, equipment longitude and latitude, etc. The date, time and device id are string data, and the others are numeric data.

#### 4.1.2. I/O Variables and Prediction Model

**Figure 7:**
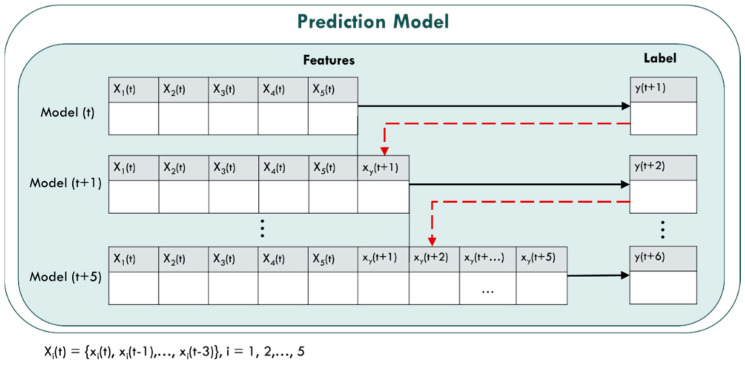
Prediction models of our method.

The prediction model of this study uses 30 min as a time period to predict the PM_2.5_ concentration value of six time periods in the next 3 h and that is *y*(t + 1), *y*(t + 2), …, *y*(t + 6). The input and output variables used by the prediction model are shown in Table 2. The model input variables are five variables: PM_2.5_, PM_10_, PM_1_, Temperature and Humidity, {*x*_1_(t), *x*_2_(t), *x*_3_(t), *x*_4_(t), *x*_5_(t)}, collected from the LASS open source community. Moreover, considering the impact of time delay, this study also includes the time in advance variable {*x_i_*(t − 1), *x_i_*(t − 2), *x_i_*(t − 3), *i* = 1, 2, …, 5}, a total of 15 in advance variables. Thus, there are 20 input variables in the Model(t) prediction model, and the output variable is the PM_2.5_ concentration predicted value *y*(t + 1). Variables of the prediction models are shown in Table 2, and six prediction models in Figure 7 are represented as:

Model(t): *y*(t + 1) = *f* [*X_i_*(t)]
Model(t + 1): *y*(t + 2) = *f* [*X_i_*(t), *x_y_*(t + 1)]
Model(t + 2): *y*(t + 3) = *f* [*X_i_*(t), *x_y_*(t + 1), *x_y_*(t + 2)]
Model(t + 3): *y*(t + 4) = *f* [*X_i_*(t), *x_y_*(t + 1), *x_y_*(t + 2), *x_y_*(t + 3)]
Model(t + 4): *y*(t + 5) = *f* [*X_i_*(t), *x_y_*(t + 1), *x_y_*(t + 2), *x_y_*(t + 3), *x_y_*(t + 4)]
Model(t + 5): *y*(t + 6) = *f* [*X_i_*(t), *x_y_*(t + 1), *x_y_*(t + 2), *x_y_*(t + 3), *x_y_*(t + 4), *x_y_*(t + 5)]
where *X_i_*(t) = {*x_i_*(t − 1), *x_i_*(t − 2), *x_i_*(t − 3), *i =* 1, 2, …, 5}
and *x_y_*(t + *j*) = *y*(t + *j*), *j =* 1, 2, …, 5

### 4.2. Evaluation

#### 4.2.1. Index of PM_2.5_

The evaluation of the predicted value of fine suspended particles (PM_2.5_) is carried out in accordance with the fine suspended particle index comparison table provided by the Environmental Protection Department of the Executive Yuan, Taiwan. There are four categories of low, medium, high, and very high in the index level classification, and they are marked in four color systems: green, yellow, red and purple. In the activity recommendations for sensitive groups, the main sensitive groups mentioned are people with heart, respiratory and cardiovascular diseases or the elderly. The detailed index table is shown in Table 3:

#### 4.2.2. Performance Index

In order to evaluate the pros and cons of the prediction model trained by the machine learning method, two evaluation metrics are used: Root mean squared error (RMSE) and coefficient of determination (R-squared, R^2^).

(1) Root mean squared error (RMSE): is the sample standard deviation of the predicted value and the actual value. Its value can be used to measure the difference between the predicted value and the actual value. The smaller the value the better the prediction performance. The calculation formula is as Equation (1):
  (1) $$\mathrm{RMSE}=\sqrt{\frac{\sum_{i=1}^{n}{\left(Actual_{i}-Predicted_{i}\right)}^{2}}{n}}$$
(2) Coefficient of determination (R2): As in Equation (2), It measures the suitability of the model to the sample value and can test the predictive ability of the model. Its value is between 0 and 1. The closer to 1, the higher the suitability of the model, and the closer to 0 indicates the lower the model fitness.
  (2) $$R^{2}=1-\frac{MSE}{VAR\left(y\right)\left(N-1\right)}=1-\frac{\sum_{i=0}^{N-1}{\left(y_{i}-{\hat{y}}_{i}\right)}^{2}}{\sum_{i=0}^{N-1}{\left(y_{i}-\bar{y}\right)}^{2}}$$

### 4.3. Experiment

**Figure 8:**
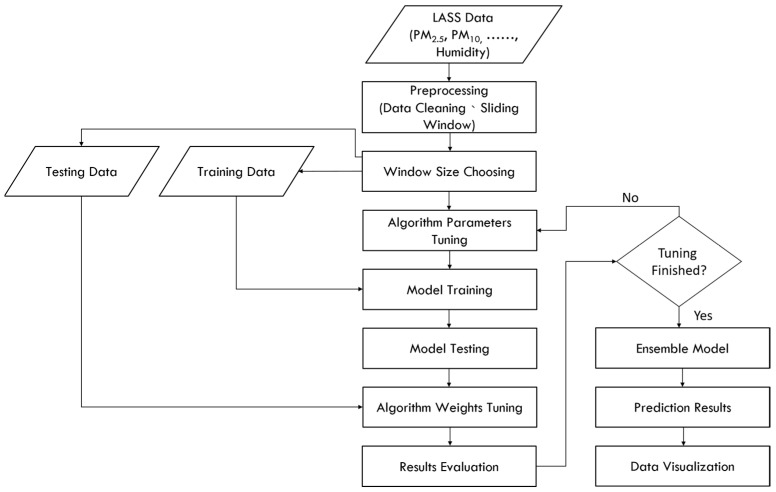
Flow chart of our experiment.

The experimental flowchart of this study is shown in Figure 8. First, collecting the LASS community data related to PM_2.5_ predictions through the data collection and processing module, and into the big data training and prediction module after data pre-processing. The Spark computing framework and ensemble machine learning will be used in the module to train the predictive model, and then use the testing dataset to test the trained model. The RMSE and R^2^ are used to evaluate the pros and cons of the prediction model. Then, the collected real-time data is used as the input of the predictive model, predict the PM_2.5_ concentration value in the next 3 h for a total of 6 time periods, so as to implement real-time PM_2.5_ concentration value prediction. Finally, in the data visualization module, a map of the PM_2.5_ concentration value distribution is generated for visualization present. The detailed description in the flowchart is as follows:

**Pre-processing**

This study uses PM_2.5_ sensor data collected from the LASS open source community. The final variables selected are PM_2.5_, PM_10_, PM_1_, Temperature and Humidity. Data cleaning has carried out null values and outlier data with null values will be deleted, in terms of outlier processing. Due to occasional sensor failure, only when data of PM_2.5_ and PM_10_ values are greater than 0 are they retained.

**Sliding window size choosing**

**Figure 9:**
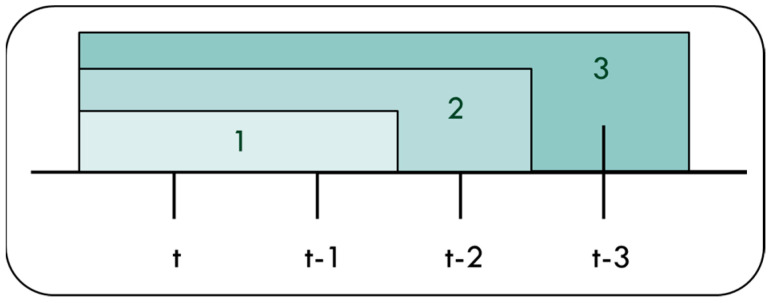
Window size of 1, 2 and 3.

Window size determination is based on the evaluation result of training as shown in Figure 9. The evaluation results of window size 1, 2 and 3 are shown in Table 4. Window size 1 includes data of {Xi(t), Xi(t − 1)}, window size 2 includes data of {Xi(t), Xi(t − 1), Xi(t − 2)} and window size 3 includes data of {Xi(t), Xi(t − 1), Xi(t − 2), Xi(t − 3)}. We can obtain that window size 3 performs better than others in Table 4. Therefore, all the input variables of prediction model will be chosen based on this result. 

**Algorithm weight turning**

The weights in the ensemble model are set by the three different algorithms through the value of the evaluation index R^2^, the weighting method of the three machine learning methods are as follows:

(3) $$w\left(zi\right)=R\left(zi\right)/\overset{3}{\underset{i=1}{\sum }}R\left(zi\right)$$

**Data visualization**

**Figure 10:**
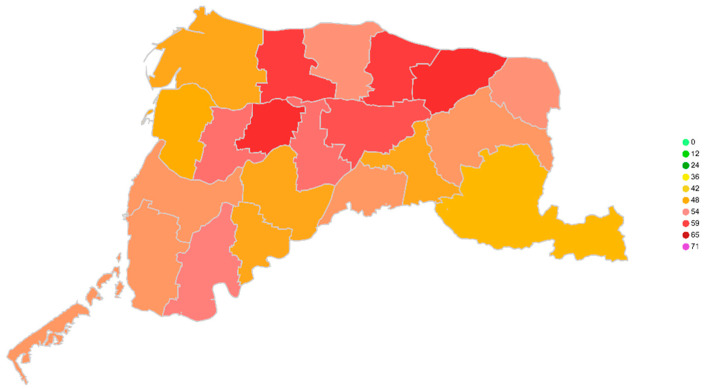
Visualization of PM_2.5_ Prediction.

This study uses the Geocoding API provided by Google to convert the latitude and longitude. In the visualization module, the prediction results of each sensor can be mapped to the area to which they belong. Figure 10 below shows the urban areas of various towns in Yunlin, Taiwan. A schematic diagram of the predicted value in a certain period of time is shown.

## 5. Experimental Results and Discussion

### 5.1. Experimental Result

**Figure 11:**
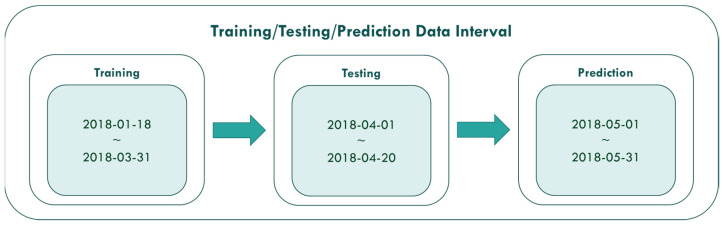
Three stages of experiment.

The PM_2.5_ concentration value prediction of this study is mainly divided into three stages of experiment. The first stage uses the data from 18 January 2018 to 31 March 2018 to conduct model training. The second stage data from 1 April to 20 April 2018 is used for model testing. Furthermore, the third stage is to use the data from 1 May 2018 to 31 May 2018 to make predictions. The data intervals used in the three stages are shown in Figure 11.

#### 5.1.1. Model Training Results

The prediction model of PM_2.5_ concentration value for six periods is built using data from 18 January 2018 to 31 March 2018. Firstly, 5,837,008 pieces of data will be used as the training data set of the model to carry out training of the six models. In order to observe whether the ensemble model performance is higher than the single regression, three algorithms of linear regression, random forest regression, gradient boost, and the integration of ensemble are compared. The effectiveness evaluation results of the four algorithms in PM_2.5_ concentration *y*(t + 1) value prediction from training data are shown in Table 5 below. The results show that the integrated ensemble model is the best among them since RMSE is the smallest and R^2^ is the largest.

Table 6 shows the effectiveness evaluation results of six Ensemble Models after training. Among them, the prediction model of PM_2.5_ concentrations after 30 min *y*(t + 1) is the best, RMSE is 10.63 and R^2^ is 0.85, while RMSE of the six models is between 10.63 and 16.32, and R^2^ is between 0.67 and 0.85.

#### 5.1.2. Model Testing Results

At the end of the training model stage, the next step will be to predict the established model. In this stage, 2,254,609 records will be used as testing data set from 1 April 2018 to 20 April 2018. The established prediction model will be input to predict the quality of the actual prediction of the model. In order to observe whether the performance of the integrated model is higher than the performance of the single model, the model performance comparison between linear regression, random forest regression and gradient lifting tree regression and integration is carried out in this phase also. Table 7 shows the results of the four algorithms in predicting the effectiveness of the PM_2.5_ concentrations after 30 min. The integrated ensemble model shows the best performance again on both indicators RMSE and R^2^. When RMSE is smaller it is better and when R^2^ is larger it is better.

Table 8 below shows the evaluation results of different Ensemble Model in the prediction phase with different time segment from *y*(t + 1) to *y*(t + 6). The results show that the performance of the model after 30 min *y*(t + 1) is the best since RMSE is 9.14, and R^2^ is 0.75.

Through the training and testing results of the model in Table 5 and Table 8, we can find that the longer the estimated PM_2.5_ value is from the present time, the easier the input variable is not related to it, which may be the reason for this situation.

**Figure 12:**
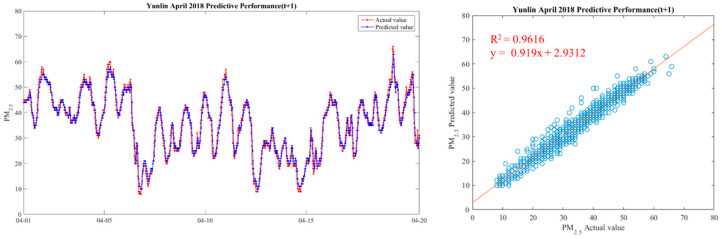
Model(t) performance.

**Figure 13:**
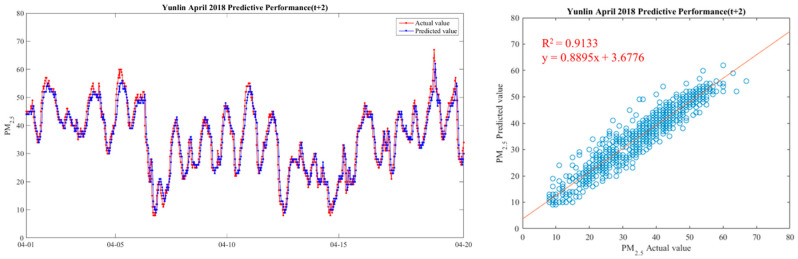
Model(t + 1) performance.

**Figure 14:**
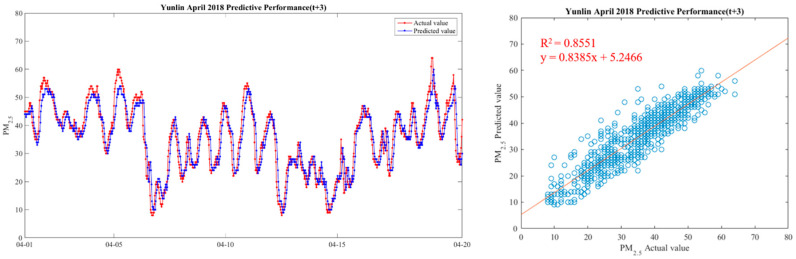
Model(t + 2) performance.

**Figure 15:**
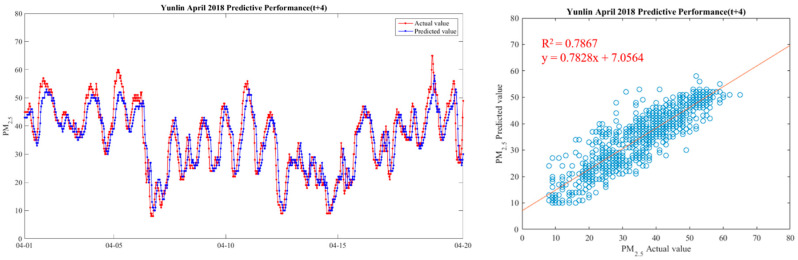
Model(t + 3) performance.

**Figure 16:**
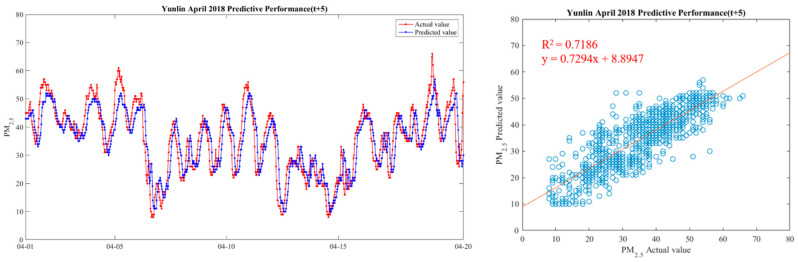
Model(t + 4) performance.

**Figure 17:**
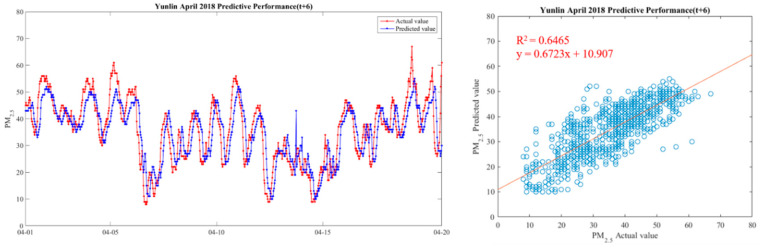
Model(t + 5) performance.

In order to make the results more intuitive, we further drew a broken line map between the actual value of PM_2.5_ concentrations and the predicted value during the period from 1 April 2018 to 20 April 2018, in order to show the predicted trend more clearly and better compare the differences between the two. We chose Yunlin County as an example in drawing, because the Ministry of Health and Welfare issued 105 years of the Republic of China. In the medical statistics of civil health insurance, Yunlin County is the leading County in the rate of chronic lower respiratory diseases per 100,000 population based on the place of residence. Thus Yunlin County is chosen as an example of the results. The drawing method of the broken line chart takes 30 min as a unit and calculates the average of the actual and predicted values of all sensors located in Yunlin County within the 30-min interval. From 1 April 2018 to 20 April 2018, there are 922 pieces of data. Finally, with 922 data points, we draw a comparison chart and regression analysis chart between the predicted value and the actual value of PM_2.5_ in six time periods in Yunlin County in Figure 12, Figure 13, Figure 14, Figure 15, Figure 16 and Figure 17 as follow:

It can be seen from the figures that the prediction model of this study has good performance, and the predicted value of PM_2.5_ concentrations is almost the same as the actual value. Among them, the Model (t) prediction model performance (R^2^ = 0.9616) in Figure 11 is the closest to the actual value, and the prediction results are as expected. As the prediction time becomes longer, the prediction performance gradually decreases. The R^2^ is a measure of the model’s predictive accuracy. This effect ranges from 0 to 1 with 1 representing complete predictive accuracy. Because R^2^ is embraced by a variety of disciplines, scholars must rely on a “rough” rule of thumb regarding an acceptable R^2^ with 0.75, 0.50 0.25, respectively describing substantial, moderate, or weak levels of predictive accuracy [46]. The highest R^2^ of the results of this study reached 0.96, which can be considered as a high-efficiency level.

### 5.2. Discussion

#### 5.2.1. Computation Time in Model Training

The experimental environment of this study is PC with Intel^®^ Core™ i7-6700HQ CPU, and the virtual machine is created through Oracle VM VirtualBox. The operating system is Ubuntu 16.04.4 LTS, memory is 8 G, and the model’s training, testing, and prediction are performed in Spark’s Local mode. Spark’s programs are written in Python. In the model training stage, Spark’s Local mode is used in a single virtual machine, and the advantages of Spark’s decentralized operation cannot be fully utilized because of the long training time. The training time of the six different ensemble models in this study is shown in Table 9. As the variable increased, the training time almost exponentially increased. One feasible solution is to move the framework onto cloud platforms such as Google Cloud Platform and level it in the cloud. The platform can rent virtual machines according to the actual needs, and flexibly adjust the number of virtual machines, memory, CPU and so on. There are also many convenient services in cloud platform. Take Google Cloud Platform as an example, the Spark experiment of this study can be carried out by Cloud Dataproc service, and data storage also provides Cloud Storage, Cloud Bigtable and other storage methods. Choices are to be made dependent on needs.

The input variables of the model are only PM_2.5_, PM_10_, PM_1_ temperature and relative humidity. The variables of rainfall, wind direction and wind speed are not used in this study, but are due to LASS Open Source Society. Data may be collected and integrated from different sources, such as the Central Meteorological Administration (CMA) and the Ministry of Communications (MOTC), which provide information on rainfall, wind direction and wind speed that can be further investigated. The data update frequency of PM_2.5_ sensor provided by LASS is five minutes, and the area of the station is not the same, therefore, synchronization also needs to be considered.

#### 5.2.2. PM_2.5_ Concentration Category Prediction

Nevertheless, in order to observe whether the higher and lower PM_2.5_ concentration values can explain the predicted results better, classified experiments were carried out according to the four categories of fine suspended particulate matter indicators provided by EPD of the Executive Yuan: low, medium, high and extremely high. The first step of the experiment is to adjust the data before and after the concentration prediction experiments, and convert the PM_2.5_ concentration values into fine ones. Spark local model was used to classify the suspended particulate matter. Random forest classification algorithm was used to classify the suspended particulate matter. The PM_2.5_ concentration category after 30 min, i.e., *y*(t + 1), was predicted. The accuracy of the experimental results was used as the evaluation index, and the results were shown in the experiment. The classification accuracy of the lower category is the most accurate, and that of the middle, high and extremely high categories decreases in sequence. This may be due to the decreasing proportion of the categories in the data. After 30 min, the overall classification accuracy of the PM_2.5_ concentration category is 0.88. The confusion matrix of PM_2.5_ concentration classification is shown in Table 10 below.

#### 5.2.3. Comparison with Other Studies

The main difference between this study and others is the prediction time period and the number of Big data sensor sites. According to the comparison table in Table 11, it can be found that other studies focus on long-term pollution predictions, while this study focuses on short-term real-time PM_2.5_ concentration predictions in the next 30 to 180 min. It is shown that this study provides more air pollution prediction information in Big data environment than others. In terms of environmental policy research, China’s clean air plan includes strengthening industrial emission standards, eliminating polluting factories and strengthening automobile emission standards. This study can also use the areas with the highest statistical concentrations to call up the industrial conditions in the vicinity of the area. To carry out an action plan, or conduct traffic guidance to reduce emissions, and increase the deployment of electric vehicles are also further options.

## 6. Conclusions

In the past, many studies have often used a long-term single learning algorithm to predict air pollution due to the high cost of monitoring stations and the limitation of computing power. Although the development of deep learning has made the accuracy rate higher in recent years, it is still a challenge in dealing with a large amount of air pollution data simultaneously. Theoretically, our study provides a prototype of a big data processing platform for practical use. This study collects PM_2.5_ sensor data from the open source community LASS, uses the Spark big data computing framework and machine learning methods, establishes six ensemble prediction models with a frequency of 30 min to predict the concentration of PM_2.5_ in Taiwan at 30 to 180 min in advance. The open source community LASS high-density sensor updates PM_2.5_ data every five minutes. These short-term PM_2.5_ concentration predictions are made by using ensemble model, which combines different time periods and other variables, and the experimental results shown an excellent result with R^2^ = 0.96 in the next 30-min. Therefore, the PM_2.5_ concentration value prediction framework of the Spark big data framework proposed in this study is highly feasible, and the prediction results can be used for reference by various departments and the public to prevent the harm caused by air pollution [48].

A large-scale public short-term air pollution prediction platform can be established for the reference of people and decision makers. As the data source area increases, the forecast range can also be expanded. Taiwan has been suffering from relatively poor air quality for a long time. Practically, this system can help decision-making units to formulate a response plan for air pollution. Such as, announcing warning areas where PM_2.5_ concentrations may be excessive, thus people there can prevent outdoor exercise or stay away from that area, etc.

The limitation in this study is the source of the model data—the open source community LASS’s high-density sensors. The sensor has the advantages of being cheap, easy to obtain by the public, and has a high deployment density. However, it is also impossible to ensure that each sensor will not fail or disconnect. The source of the data comes from the public community, and there may be data errors. However, according to the experiment of Taiwan’s Environmental Protection Agency, the trend of concentration changes is similar to that of the official monitoring station, and Taiwan’s open data platform also includes relevant sensor data. Therefore, the need to ensure the integrity of the data is the difficulty encountered in this study. In addition, the model input variables in this study only have five variables, which do not include other variables related to PM_2.5_ such as rainfall, wind direction, and traffic data. In the future, one can consider how to integrate these data into the model in real time to improve the integrity of the PM_2.5_ prediction model.

In environmental and city policy research, China’s clean air actions include strengthening industrial emission standards, eliminating polluting factories and strengthening vehicle emission standards [55,56]. This study can also use the areas with the highest statistical concentration to investigate the industrial conditions for future action plans, or conduct traffic guidance to reduce emissions. In addition, increasing the deployment of electric vehicles is another option to consider.

In future, we plan to explore the performance of the techniques using other ensemble methods and to study other factors that affect air pollution. Future work will also focus on forecasting image-based air quality using a convolutional deep learning neural network. Nevertheless, the machine learning strategies and AI-based detection technique of smart air quality monitoring may lead to applications in future studies of the IoT network.

The main input variables of the prediction model of this study are only five variables, PM_2.5_, PM_10_, PM_1_, temperature and relative humidity limited by sensor. If we want to improve the prediction, one may consider including other variables such as rainfall, wind direction and wind speed. Since the LASS open source community sensor currently does not provide all these variables, it may be necessary to collect and integrate data from different sources.

The possible framework for the combination of the mobile data and station data could contribute to air quality prediction by the data resolution of time. The accurate spatial division and complete temporal analysis of the original data would further improve the scientific capacity and accuracy of the prediction, in which advanced knowledge or problem-solving abilities are required.

## Figures and Tables

**Table 1:** Related studies in Air Pollution.

Field	Methods	Air Pollutants	Authors
Variables related to air pollution	Grey system correlation analysis, linear regression	PM_2.5_	Lu et al. [25]
	Back Propagation Neural Network	PM_2.5_	Ni et al. [26]
	Multifractal descended cross-correlation analysis, (MF-DCCA)	PM_2.5_	Zhang et al. [24]
Air pollutant concentration prediction	Neural Network	CO\SO_2_\PM_10_	Kurt & Oktay [6]
		NO_2_	Russo, Raischel, & Lind [36]
		PM_2.5_\PM_10_	Voukantsis et al. [27]
		PM_2.5_	Yao et al. [28]
		PM_2.5_	Shah et al. [37]
	Hidden Markov Model	PM_2.5_	Dong et al. [38]
		PM_2.5_	Sun et al. [29]

**Table 2:** Variables of the prediction models.

Data Source	Input/Output Variables	Contents	Authors
LASS Community	*y*(t + 1)	PM_2.5_(t + 1)	Voukantsis et al. [27]; Sun et al. [29]; Polichetti et al. [48]; Li et al. [7]
	*y*(t + 2)	PM_2.5_(t + 2)	
	*y*(t + 3)	PM_2.5_(t + 3)	
	*y*(t + 4)	PM_2.5_(t + 4)	
	*y*(t + 5)	PM_2.5_(t + 5)	
	*y*(t + 6)	PM_2.5_(t + 6)	
	*x_y_*(t + 1)	PM_2.5_ (t + 1)	
	*x_y_*(t + 2)	PM_2.5_(t + 2)	
	*x_y_*(t + 3)	PM_2.5_(t + 3)	
	*x_y_*(t + 4)	PM_2.5_(t + 4)	
	*x_y_*(t + 5)	PM_2.5_(t + 5)	
	*x*_1_(t)	PM_2.5_(t)	
	*x*_1_(t − 1)	PM_2.5_(t − 1)	
	*x*_1_(t − 2)	PM_2.5_(t − 2)	
	*x*_1_(t − 3)	PM_2.5_(t − 3)	
	*x*_2_(t)	PM_10_(t)	Voukantsis et al. [27]; Polichetti et al. [48]; Li et al. [7]
	*x*_2_(t − 1)	PM_10_(t − 1)	
	*x*_2_(t − 2)	PM_10_(t − 2)	
	*x*_2_(t − 3)	PM_10_(t − 3)	
	*x*_3_(t)	PM_1_(t)	Buczyńska et al. [53]; Kwon, Jeong, Park, Kim, Cho, [54]
	*x*_3_(t − 1)	PM_1_(t − 1)	
	*x*_3_(t − 2)	PM_1_(t − 2)	
	*x*_3_(t − 3)	PM_1_(t − 3)	
	*x*_4_(t)	Temp.(t)	Voukantsis et al. [27]; Sun et al. [29]
	*x*_4_(t − 1)	Temp.(t − 1)	
	*x*_4_(t − 2)	Temp.(t − 2)	
	*x*_4_(t − 3)	Temp.(t − 3)	
	*x*_5_(t)	Humidity(t)	Voukantsis et al. [27]; Sun et al. [29]; Polichetti et al. [48];
	*x*_5_(t − 1)	Humidity(t − 1)	
	*x*_5_(t − 2)	Humidity(t − 2)	
	*x*_5_(t − 3)	Humidity(t − 3)	

**Table 3:** Index of PM_2.5_.

Index Classification	Low	Medium, High	High	Very High
PM_2.5_ (μg/m^3^)	0–35	36–53	54–70	≥71
Activity suggestion for General public	Can go out normally	Can go out normally	If feel unwell, should consider reducing outdoor activities	If feel unwell, should reduce outdoor activities and physical exertion

**Table 4:** Performance of window size.

		1	2	3
	
RMSE		10.65	10.63	10.27
R^2^		0.80	0.80	0.81

**Table 5:** Evaluation results of different algorithm from training data.

Evaluation Index		
Algorithm	RMSE	R^2^
linear regression	11.40	0.83
Random Forest	10.90	0.85
Gradient Boost	11.12	0.84
Ensemble	10.63	0.85

**Table 6:** Evaluation results of different Ensemble Model from training data.

Evaluation Index		
Time segment	RMSE	R^2^
*y*(t + 1)	10.63	0.85
*y*(t + 2)	12.18	0.81
*y*(t + 3)	13.41	0.77
*y*(t + 4)	14.77	0.73
*y*(t + 5)	15.46	0.70
*y*(t + 6)	16.32	0.67

**Table 7:** Effectiveness Evaluation Results of Prediction using different algorithm.

Evaluation Index		
Algorithm	RMSE	R^2^
Linear regression	9.18	0.75
Random Forest	9.38	0.73
Gradient Boost	11.82	0.58
Ensemble Model	9.14	0.75

**Table 8:** Evaluation Results of Prediction in next 3 h.

Evaluation Index		
Time segment	RMSE	R^2^
*y*(t + 1)	9.14	0.75
*y*(t + 2)	10.01	0.68
*y*(t + 3)	10.76	0.64
*y*(t + 4)	11.88	0.56
*y*(t + 5)	12.97	0.50
*y*(t + 6)	13.79	0.41

**Table 9:** PM2.5 Predictive Model Training Time.

Time Segment	Training Time (s)
*y*(t + 1)	1998.43
*y*(t + 2)	3226.57
*y*(t + 3)	6755.18
*y*(t + 4)	10,066.60
*y*(t + 5)	15,564.86
*y*(t + 6)	21,876.73

**Table 10:** Classification results of confusion matrix.

	Predictive Value					
Actual value		Low	Medium	High	Extremely high	total
	Low	332,951	19,195	185	12	352,343
	Medium	17,599	110,262	5318	119	133,298
	High	426	10,636	15,990	328	27,380
	Extremely high	310	1590	6697	669	9266
	total	351,286	141,683	28,190	1128	522,287

**Table 11:** Comparison with other studies.

Author	Method	Time Seg.	Station	Parti.	Index	Value
Dong et al. [38]	Hidden semi-Markov	Next 24 h	12	PM_2.5_	R^2^	-
					RMSE	-
Kurt & Oktay [6]	neural networks (GFM_NN)	3 days in advance	10	SO_2_, CO, PM_10_	R^2^	-
					RMSE	-
Kwon et al. [54]	Universal kriging models	Annual average	277	NO_2_, PM_10_	R^2^	0.46
					RMSE	6.31
Pak et al. [46]	CNN-LSTM model	Next 24 h	384	PM_2.5_	RMSE	2.875
					MAE	2.117
					MAPE	0.037
Ours	Ensemble Big data	Next 30 to 180 min.	2000 up	PM_2.5_	R^2^	0.75
					RMSE	9.14

## Data Availability

Data sharing not applicable.
